# KIF20A as a potential biomarker of renal and bladder cancers based on bioinformatics and experimental verification

**DOI:** 10.18632/aging.204736

**Published:** 2023-05-24

**Authors:** Haoyuan Wang, Xiaopeng Ma, Sijie Li, Jianzhi Su, Bo Fan, Bin Liu, Xiaochen Ni

**Affiliations:** 1 Department of Urology Surgery, The Fourth Hospital of Hebei Medical University, Shijiazhuang 050000, Hebei, P.R. China; 2 School of Basic Medical Sciences, Hebei Medical University, Shijiazhuang 050000, Hebei, P.R. China

**Keywords:** renal cancer, KIF20A, bladder cancer, potential biomarker, bioinformatics

## Abstract

Background: Bladder cancer (BC) is a malignant tumor that occurs in the bladder wall and often appears in elderly individuals. Renal cancer (RC) arises from the renal tubular epithelium, but its molecular mechanism remains unclear.

Methods: We downloaded RC datasets (GSE14762 and GSE53757) and a BC dataset (GSE121711) to screen differentially expressed genes (DEGs). We also performed weighted gene coexpression network analysis (WGCNA). We created a protein-protein interaction (PPI) network and performed functional enrichment analysis, such as gene set enrichment analysis (GSEA). Heatmaps were made for gene expression. Survival analysis and immunoinfiltration analysis were performed. Comparative toxicogenomics database (CTD) analysis was performed to find the relationship between disease and hub genes. Western blotting was performed to verify the role of KIF20A in apoptosis.

Results: A total of 764 DEGs were identified. The GSEA showed that the DEGs were mainly enriched in organic acid metabolism, drug metabolism, mitochondria, and metabolism of cysteine and methionine. The PPI network in GSE121711 showed that KIF20A was a hub gene of renal clear cell carcinoma. Where the expression level of KIF20A was higher, the prognosis of patients was worse. CTD analysis showed that KIF20A was associated with inflammation, proliferation, and apoptosis. KIF20A expression in the RC group was upregulated, as shown by western blotting. The core proteins (including pRB Ser 780, CyclinA, E2F1, CCNE1, and CCNE2) in the pRB Ser 780/CyclinA signaling pathway were also upregulated in the RC group.

Conclusions: KIF20A might be a novel biomarker for researching renal and bladder cancers.

## INTRODUCTION

Bladder cancer (BC) is a frequently occurring malignant disease [1, 2]. Its incidence has increased in many countries, especially in Europe. Most BCs (90%) are urothelial cancer [3, 4]. The mortality rate for BC is high, with a 5-year survival rate of more than 80% in the early stage, approximately 50% in the middle stage, and less than 20% in the late stage [5]. Hematuria may occur in the early stage of BC, but it may not be obvious in some patients [6]. In China, the most obvious causes are smoking and occupational exposure to aromatic amines [7, 8].

Renal cell carcinoma (RCC) is a urinary system tumor, and the vast majority of malignant renal tumors are RCC [9]. Patients with RCC are concentrated between 40 and 55 years of age, and its incidence is higher in the United States and Europe than in Asia [10]. However, the pathogenesis of BC and RCC is unknown.

As an important part in the development of life science studies, bioinformatics has been at the forefront of life science and technology research. In recent years, China's biotechnology has developed by leaps and bounds, and bioinformation resources have also grown dramatically. Bioinformatics technology is an interdisciplinary subject of biology. Bioinformatics reveals the biological significance represented by big data, which is a bridge between data and the clinic. Represented by the analysis and reporting of gene detection data, bioinformatics plays an important role in tumor treatment [11, 12].

KIF20A is abnormally expressed in multiple cancer tissues. However, its relationship with BC and RCC is not clear. This paper presents an analysis of renal cell carcinoma to identify key genes and verify whether KIF20A affects RCC and BC.

## MATERIALS AND METHODS

### RCC and BC datasets

The search was conducted in GEO from December 31, 2000, to October 31, 2023, and the keywords for searching were “renal cancer”, “bladder cancer”, “clear cell renal cell carcinoma”, and “ccRCC”. The entry type was “series”. The study type was “expression profiling by array”. “*Homo sapiens*” was the selected item. We obtained RCC datasets GSE14762 [13] and GSE53757 [14] and BC dataset GSE121711 from GEO using configuration files generated from GPL4866, GPL570, and GPL17586. GSE14762 consisted of 10 RCC and 12 normal tissues, GSE53757 consists of 72 RCC and 72 normal tissues, and GSE121711 consisted of 8 BC and 10 normal tissues.

### Batch effect removal and screening of DEGs

The inSilicoMerging package in R was used to merge the matrix. The removeBatchEffect function was used to remove batch effects. "Limma" was used to identify the DEGs. The DEGs were identified for tumor vs. control. The cutoff criteria for DEGs were p<0.05 and a fold change >3. The results were visualized using a volcano plot.

### Weighted gene co-expression network analysis (WGCNA)

The first step was to preprocess the gene expression data to remove any technical variations. The next step was to construct a pairwise correlation matrix between all pairs of genes in the dataset. The correlation matrix was then transformed into an adjacency matrix using a soft-thresholding power function to emphasize strong correlations while downweighting weak correlations. Once the adjacency matrix was computed, modules or clusters of highly correlated genes were identified using hierarchical clustering or other clustering algorithms. These modules were assigned different colors for easy visualization. Module preservation analysis was conducted as follows: To ensure the robustness of the identified modules, preservation analysis was performed to evaluate the stability and reproducibility of the modules across different datasets or sample sizes. Module annotation was conducted as follows: After identifying the modules, the biological significance of each module was assessed by performing functional enrichment analysis to identify overrepresented biological pathways, Gene Ontology (GO) terms, or other annotation terms. The final step was to correlate the identified modules with the external traits of interest.

### PPI network

STRING was performed to build a PPI network with the DEGs. Cytoscape software was used for visualization, and MCODE (https://cytoscape.org/) identified the hub module.

### Functional enrichment analysis

We used the R package clusterProfiler (version 3.14.3) for enrichment analysis. Enrichment analysis was performed using the Metascape database. Functional enrichment analysis is a computational method used to determine whether a set of genes or proteins is enriched for particular biological functions, pathways, or processes. This type of analysis is commonly used in genomics and proteomics research to gain insights into the biological mechanisms underlying a set of experimental observations.

### GSEA

The gene expression data were normalized, and genes with low expression were filtered out to reduce noise. A set of gene sets was defined, usually based on prior knowledge of biological pathways or molecular functions. Enrichment scores were calculated. Statistical significance testing was conducted as follows: The significance of the enrichment scores was assessed by permutation testing, comparing the observed scores to the distribution of scores obtained by randomly permuting the sample labels. This study aimed to determine the differential gene list input into the KEGG REST API.

### Heatmap of gene expression

The R package heatmap was used to make a heatmap of the degree of expression of core genes in GSE14762 and GSE53757, visually displaying expression differences of core genes among RCC, BC and normal tissue.

### Survival analysis

The GEPIA database (http://gepia.cancer-pku.cn/) derives its data from the TCGA (https://www.cancer.gov/ccg/research/genome-sequencing/tcga) and GTEx databases. We used GEPIA for survival analysis.

### Immunoinfiltration analysis

The LM22 gene signature was used to define 22 immune cell subtypes. We applied an integrated bioinformatics approach and used the CIBERSORT (http://CIBERSORT.stanford.edu/) software package to analyze the batch-corrected merged matrices from GSE14762 and GSE53757. We selected samples with sufficient confidence levels based on a cutoff of p < 0.05.

### Comparative toxicogenomics database analysis

Key genes were input into CTD (http://ctdbase.org/), the diseases associated with key genes were found, and a bar graph of gene expression differences was created using Excel.

### Verification of the role of KIF20A in apoptosis by Western blotting

Normal renal cells (293 [HEK-293]) and renal cancer cells (Caki-1) were obtained from the National Biomedical Experimental Cell Resource Bank (Beijing, China). Cells were divided into 4 groups: normal renal cancer cells (Con), renal cancer cells (RC), RC/KIF20A-knockdown group (RC/KIF20A-KO), and RC/KIF20A-overexpression group (RC/KIF20A-OE). Sample preparation was conducted as follows: First, the protein of interest was extracted and purified from the sample. This involved homogenization, cell lysis, and/or chromatography, depending on the sample type. Protein separation was conducted as follows: Extracted protein was separated based on molecular weight using SDS-PAGE (sodium dodecyl sulfate-polyacrylamide gel electrophoresis). Protein transfer was conducted as follows: After separation, the proteins were transferred from the gel to a solid support, either nitrocellulose or a PVDF (polyvinylidene fluoride) membrane, using electroblotting. During this step, proteins were immobilized on the membrane. Blocking was conducted as follows: To prevent nonspecific binding of the primary antibody, the membrane was incubated with blocking solution, either 5% nonfat dry milk or 1% BSA in Tris-buffered saline (TBS) with 0.1% Tween-20, for approximately one hour at room temperature or overnight at 4° C. Primary antibody incubation was conducted as follows: The membrane was incubated with a primary antibody specific to the protein of interest diluted in blocking solution for approximately one hour at room temperature or overnight at 4° C. Secondary antibody incubation was conducted as follows: After washing, the membrane was incubated with a secondary antibody conjugated to an enzyme such as horseradish peroxidase (HRP) or alkaline phosphatase (AP), which recognizes and binds to the primary antibody. The membrane was then washed again to remove any unbound secondary antibody. Signal detection was conducted as follows: The membrane was treated with substrate solution specific to the enzyme conjugated to the secondary antibody. This produced a chemiluminescent or chromogenic signal that could be detected using X-ray film or a specialized imaging system. Data analysis was conducted as follows: Since the intensity of the signal is proportional to the amount of protein present in the sample, the signal intensity was quantified using image analysis software, and the results were compared between different samples or treatments.

### Availability of data and materials

The datasets generated and/or analyzed during the current study are available from the corresponding author on reasonable request.

## RESULTS

### Differential gene expression analysis

**Figure 1:**
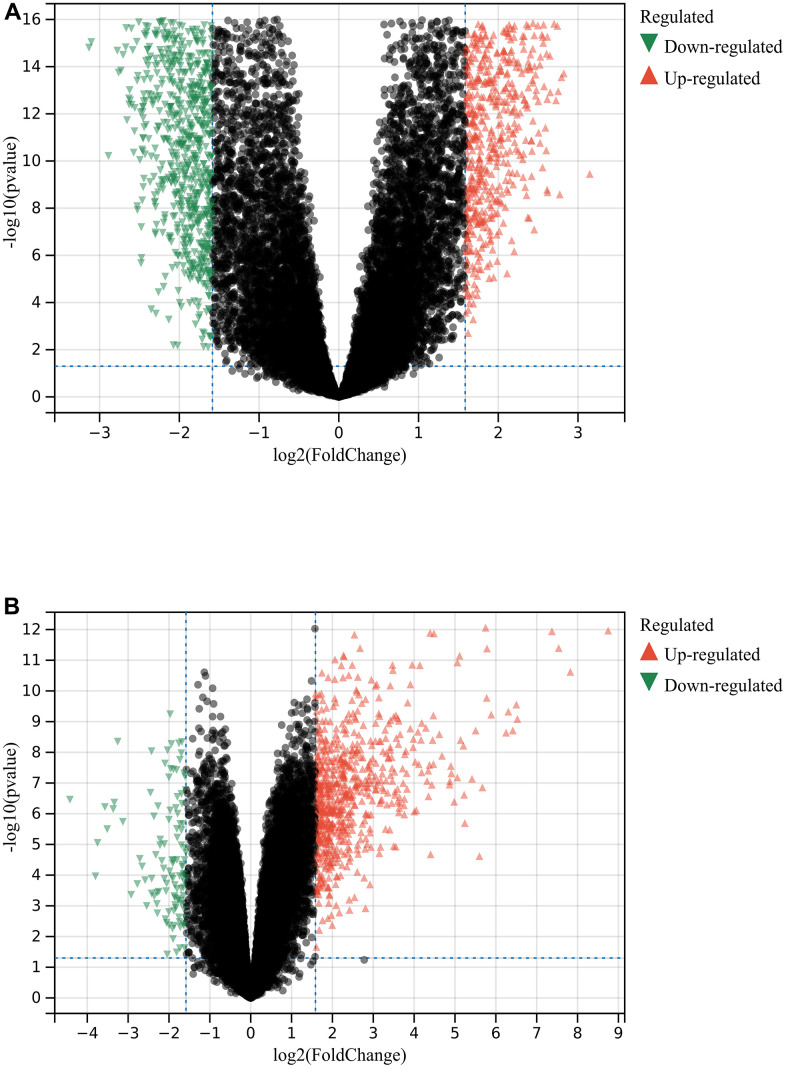
**Differential gene analysis.** (**A**) DEGs in GSE14762 and GSE53757. (**B**) DEGs in GSE121711.

A total of 1720 DEGs were found from the batch-corrected merged matrices of GSE14762 and GSE53757, according to preset cutoff values (Figure 1A). A total of 764 DEGs were identified from GSE121711 (Figure 1B).

### Functional enrichment analysis

### GSEA

**Figure 2:**
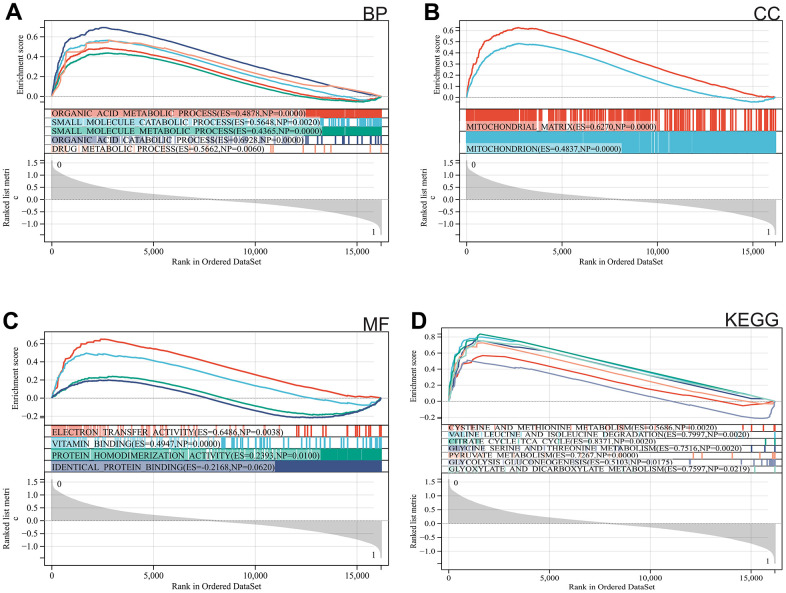
**Functional enrichment analysis.** (**A**) BP, (**B**) CC, (**C**) MF, (**D**) KEGG analysis by GSEA.

We used GSEA to perform enrichment analysis of the entire genome. The major enriched pathways were identified as organic acid metabolism, drug metabolism, mitochondria, and metabolism of cysteine and methionine (Figure 2A–2D).

### Metascape enrichment analysis

**Figure 3:**
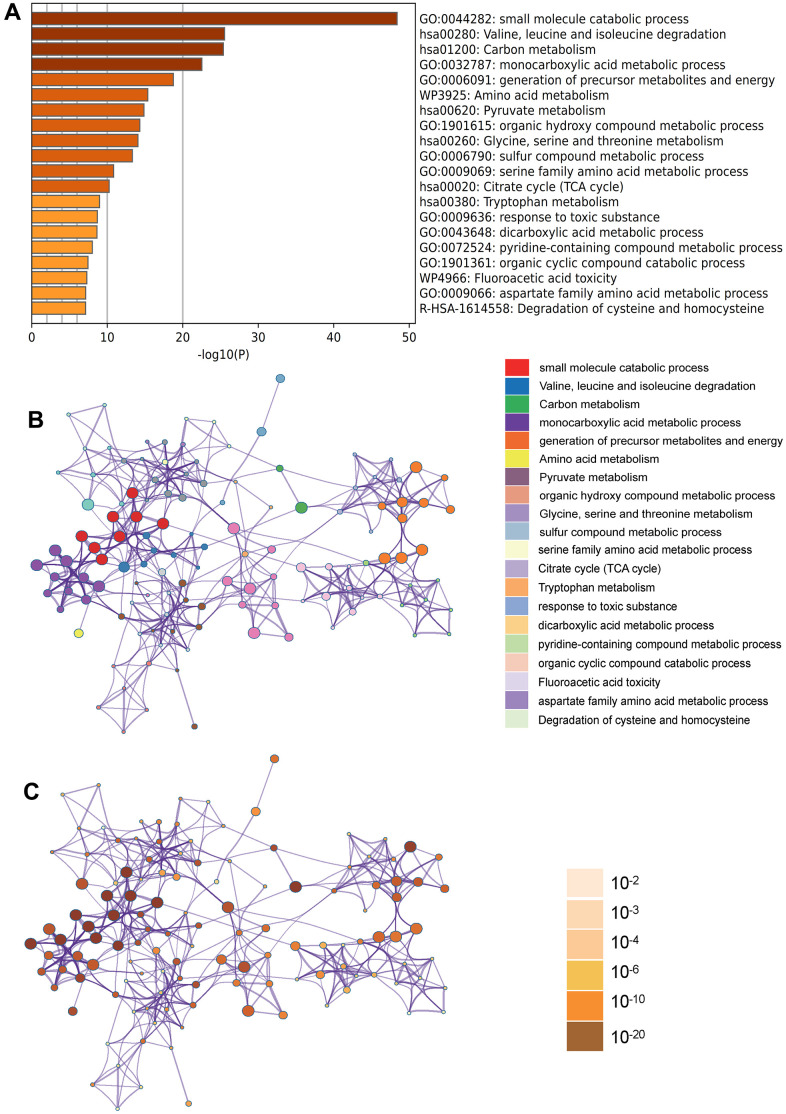
**Metascape enrichment analysis.** (**A**) Heatmap of enriched terms across input differently expressed gene lists, colored by p values, via Metascape; (**B**) Network of enriched terms colored by cluster identity, where nodes that share the same cluster identity are typically close to each other; (**C**) Network of enriched terms colored by p value, where terms containing more genes tend to have a more significant p value.

**Figure 4:**
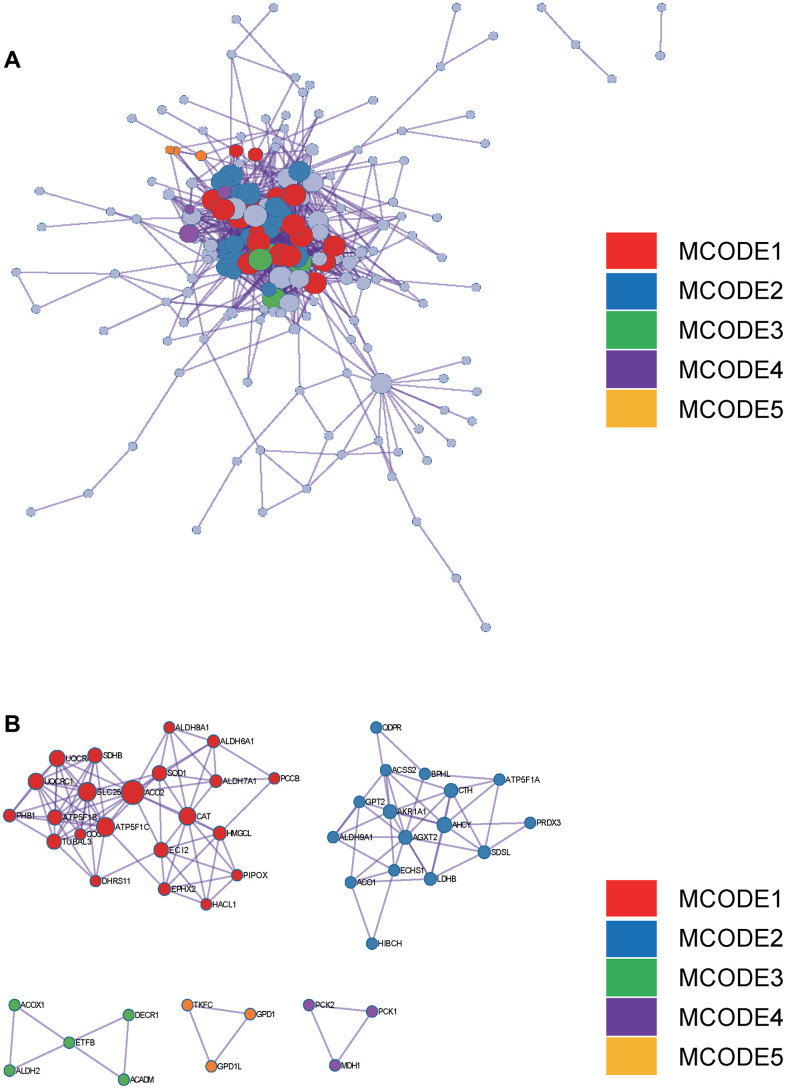
**Metascape enrichment analysis.** (**A**) PPI network drawn by Metascape. (**B**) The screened core gene group.

In the Metascape enrichment analysis, the GO enrichment analysis results showed processes such as small molecule catabolic processes, carbon metabolism, and organic hydroxy compound metabolic processes (Figure 3A). Additionally, we also output the enriched network colored by enrichment items and p values (Figures 3B, 3C, 4A, 4B) to visually represent the associations and confidence levels of each enrichment analysis.

### WGCNA

**Figure 5:**
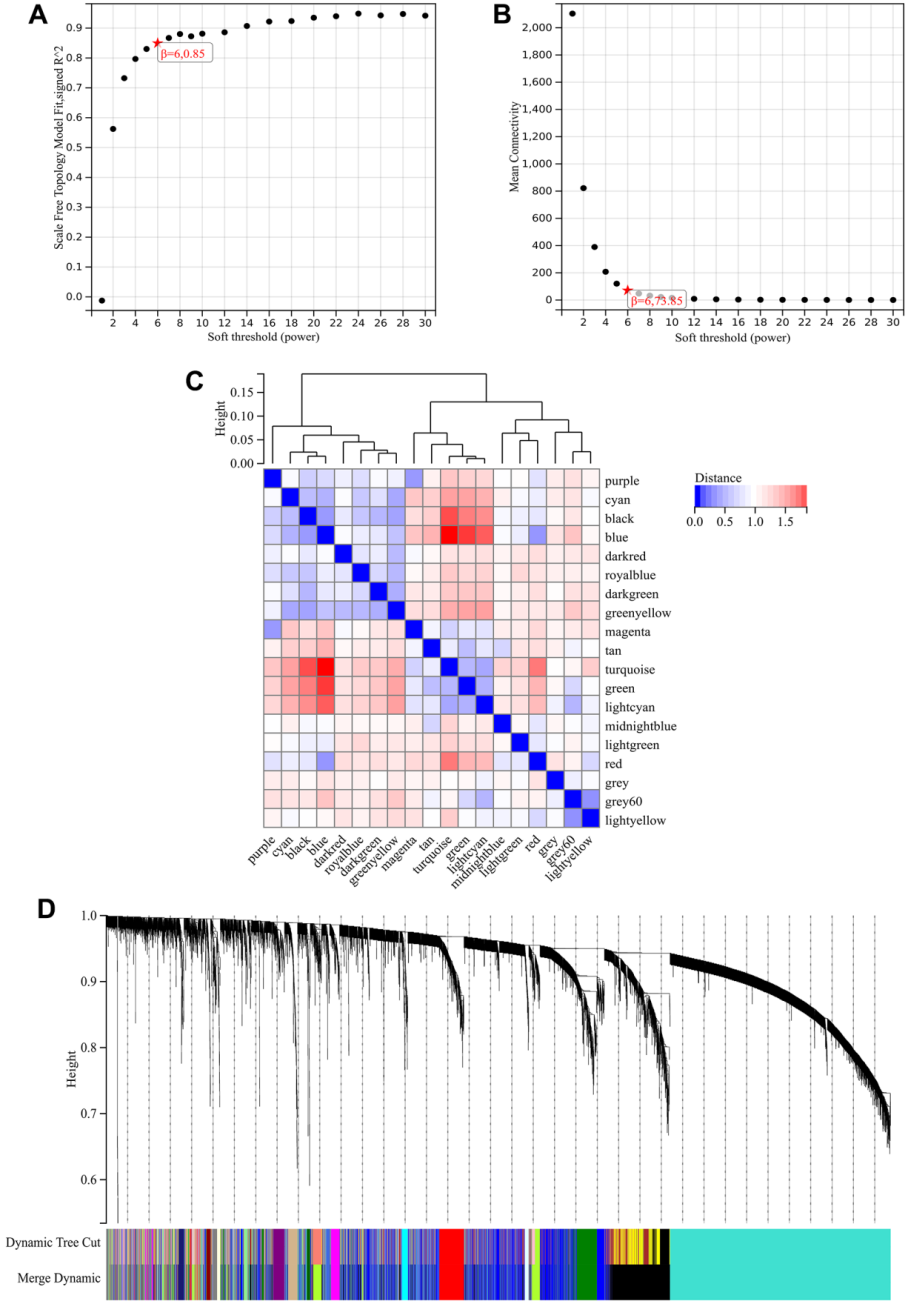
**WGCNA.** (**A**) β=6, 0.85. (**B**) β=6, 73.85. (**C**, **D**) A hierarchical clustering tree of all genes was constructed, important modules were generated, and the interactions between these modules were then analyzed.

**Figure 6:**
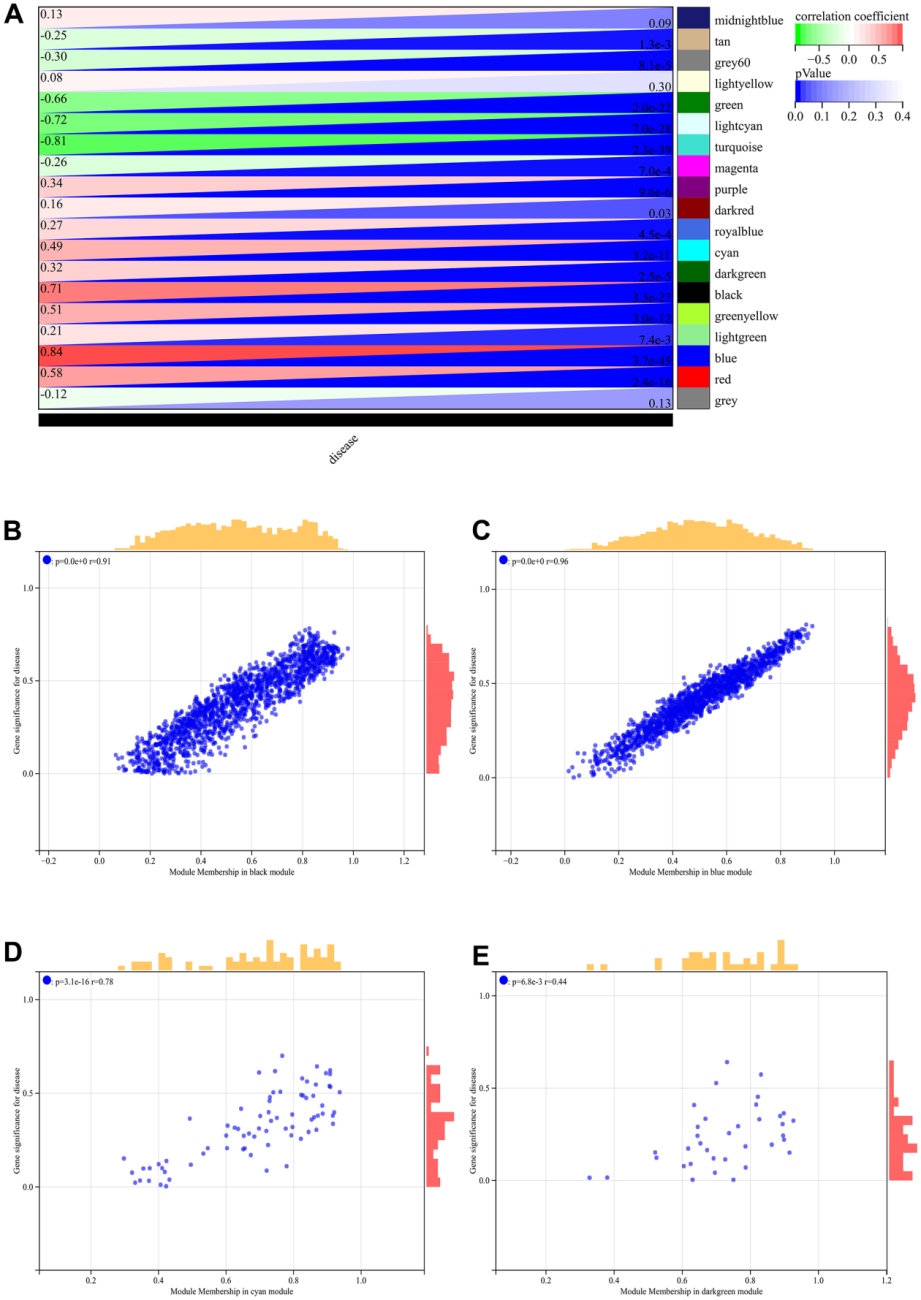
**WGCNA.** (**A**) Module-to-phenotypic correlation heatmap. (**B**–**E**) GS vs. MM correlation scatterplot.

**Figure 7:**
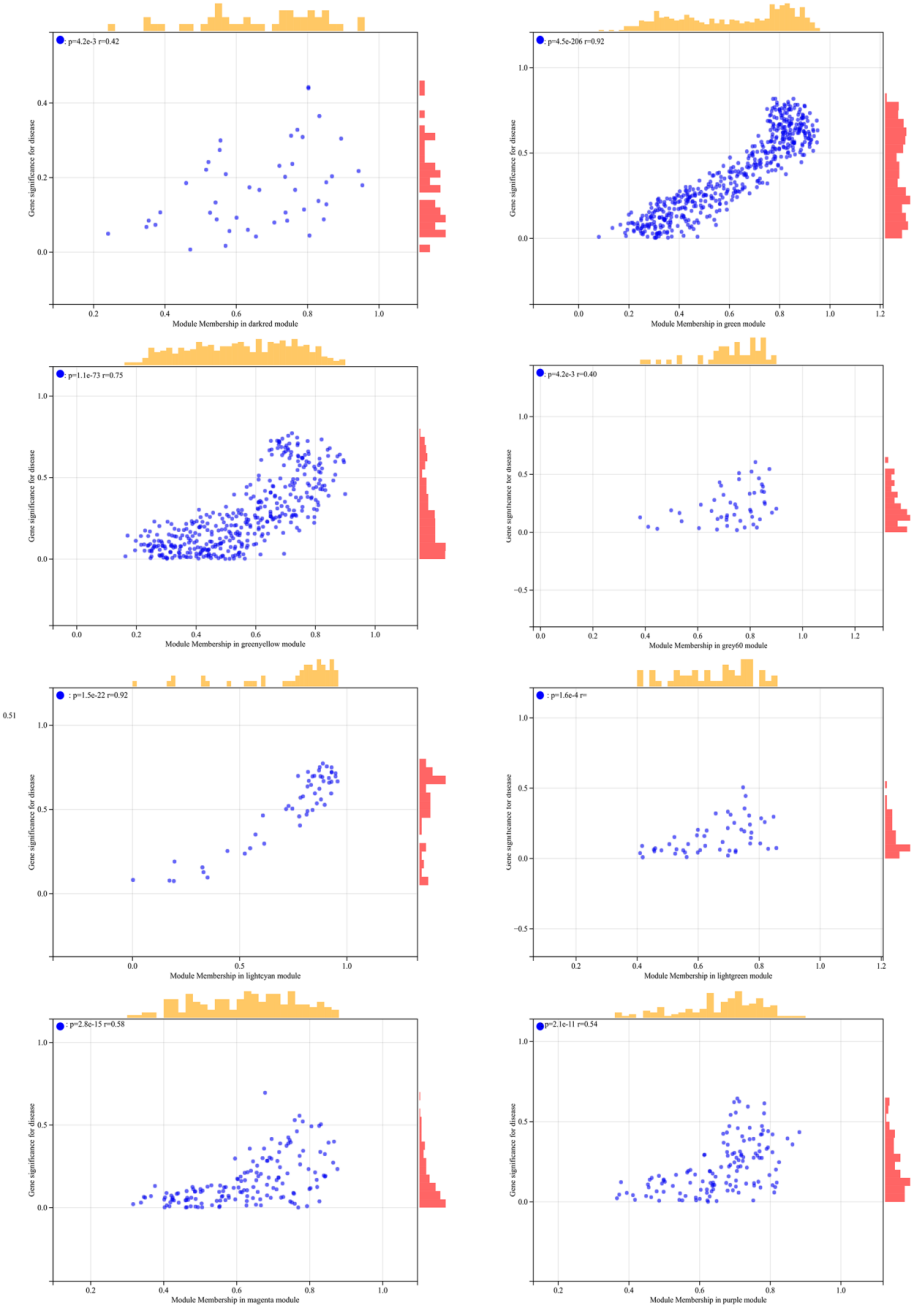
The correlation scatterplot in WGCNA.

**Figure 8:**
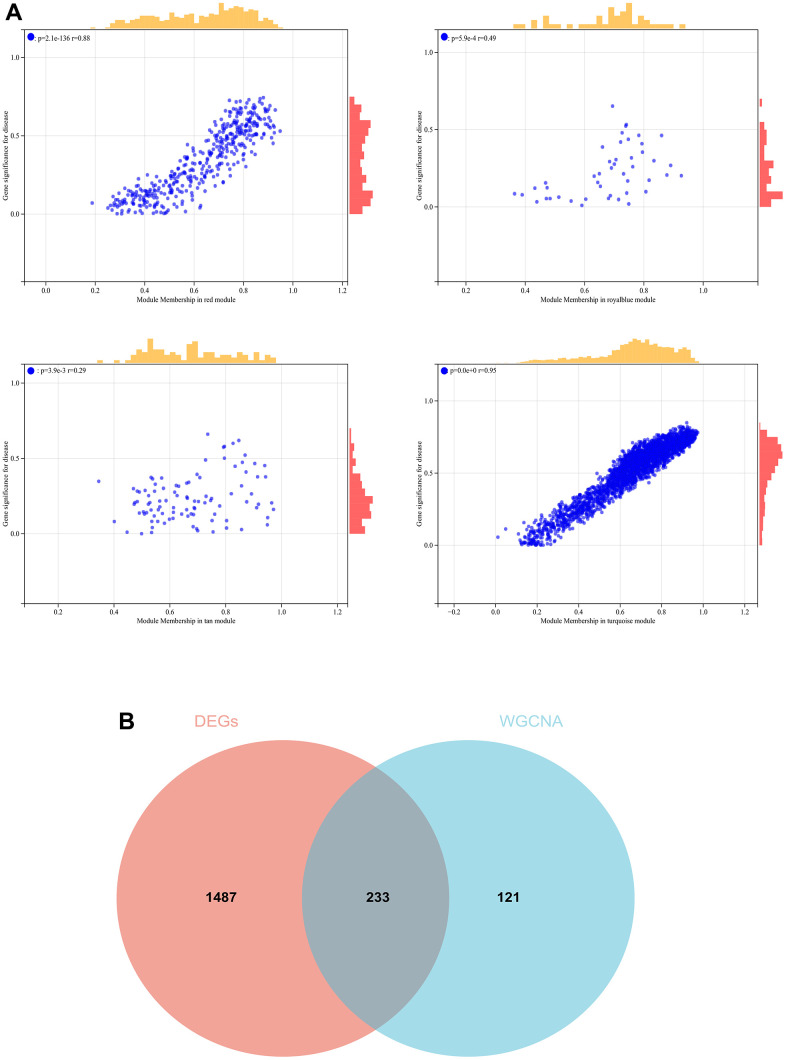
**The conjoint analysis for the WGCNA and the DEGs.** (**A**) Correlation analysis by WGCNA. (**B**) A Venn diagram was generated by intersecting the differentially expressed genes (DEGs) selected by WGCNA and DEGs and was then used to create and analyze a protein-protein interaction network.

In the WGCNA, the soft threshold power was set to 6 (Figure 5A, 5B), and interactions were found between these modules (Figure 5C, 5D). A module-phenotype correlation heatmap (Figure 6A) and scatterplots of the GS versus MM correlation of relevant hub genes (Figures 6B–6E, 7, 8A) were generated.

We also generated a Venn diagram for the WGCNA with differential genes screened as DEGs and assessed the intersection (Figure 8B).

### PPI network

**Figure 9:**
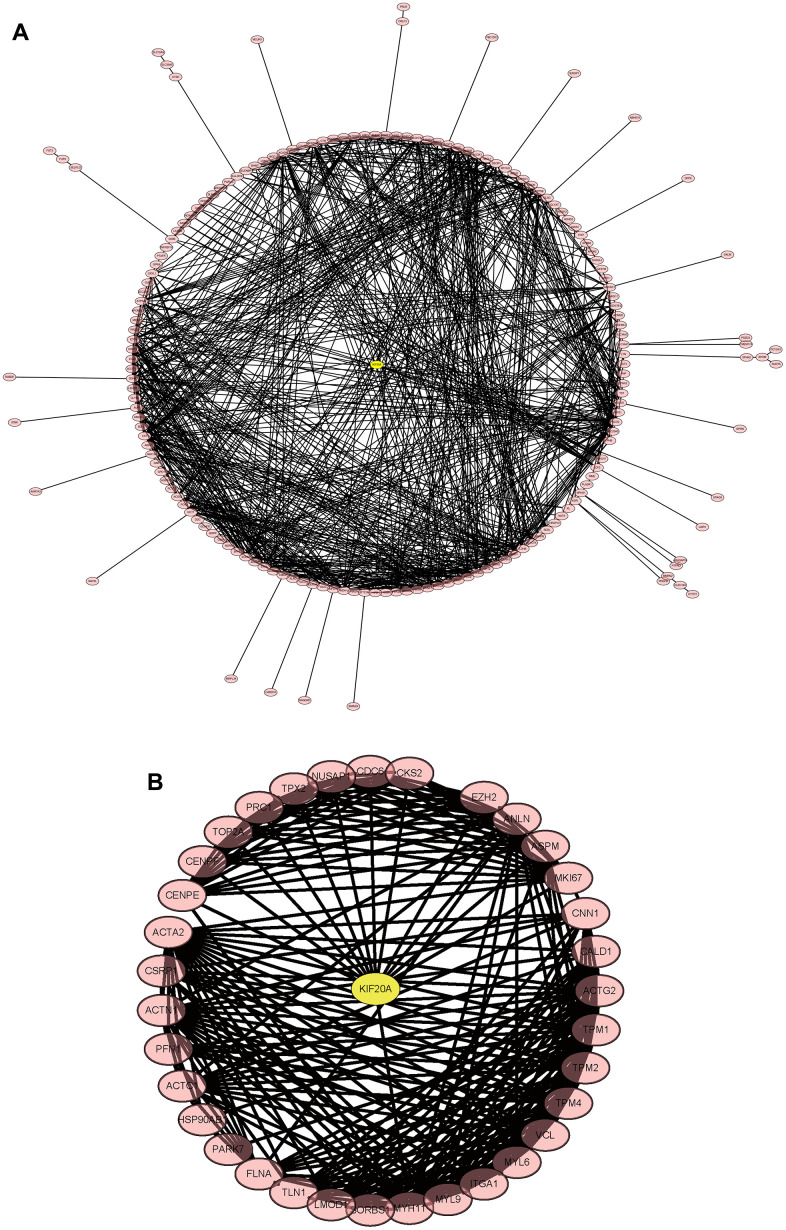
**Construction and analysis of the protein-protein interaction (PPI) network.** (**A**) GSEGSE14762, GSE53757. (**B**) GSE121711.

The merged matrix results of GSE14762 and GSE53757 are shown in Figure 9A. The interaction network of GSE121711 shows KIF20A as the core gene (Figure 9B).

### Survival analysis

**Figure 10:**
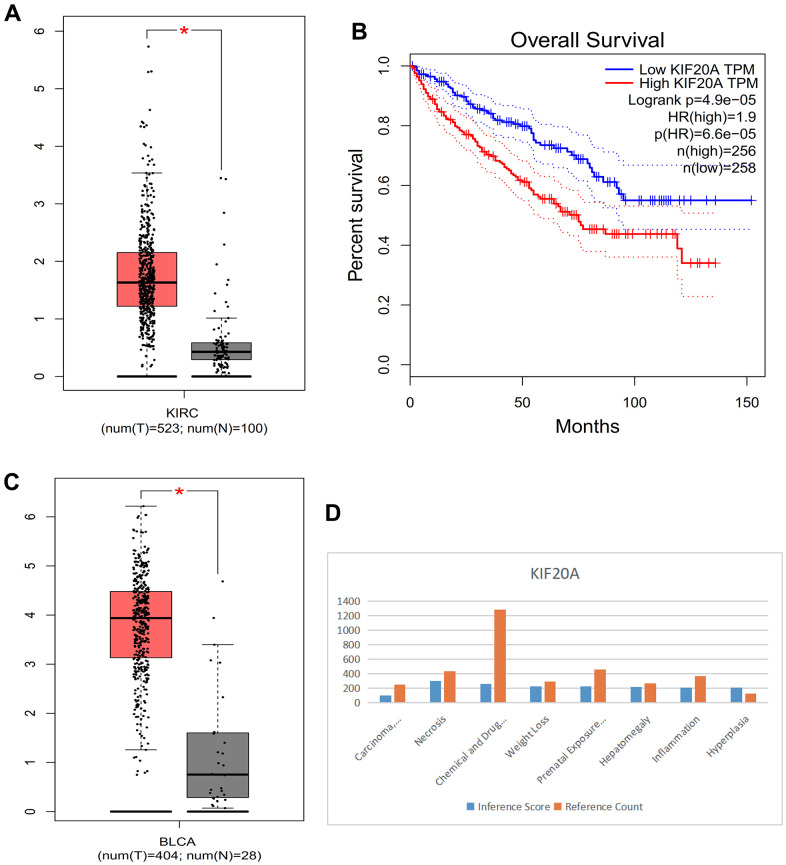
(**A**) Box plot of KIRC. (**B**) Survival curve. (**C**) Box plot of BLCA. (**D**) Histogram of CTD.

Compared with normal kidney tissue, KIF20A in RCC had high expression (Figure 10A). Patients with lower KIF20A expression had good overall survival (Figure 10B). Compared with normal bladder tissue, in BC tissue KIF20A was highly expressed (Figure 10C). High KIF20A gene expression is associated with a worse prognosis.

### CTD analysis

We found that the KIF20A gene is linked to liver cell carcinoma, necrosis, chemical- and drug-induced liver damage, weight loss, delayed effects of prenatal exposure, liver enlargement, inflammation, and proliferation (Figure 10D).

### Gene expression heatmap

**Figure 11:**
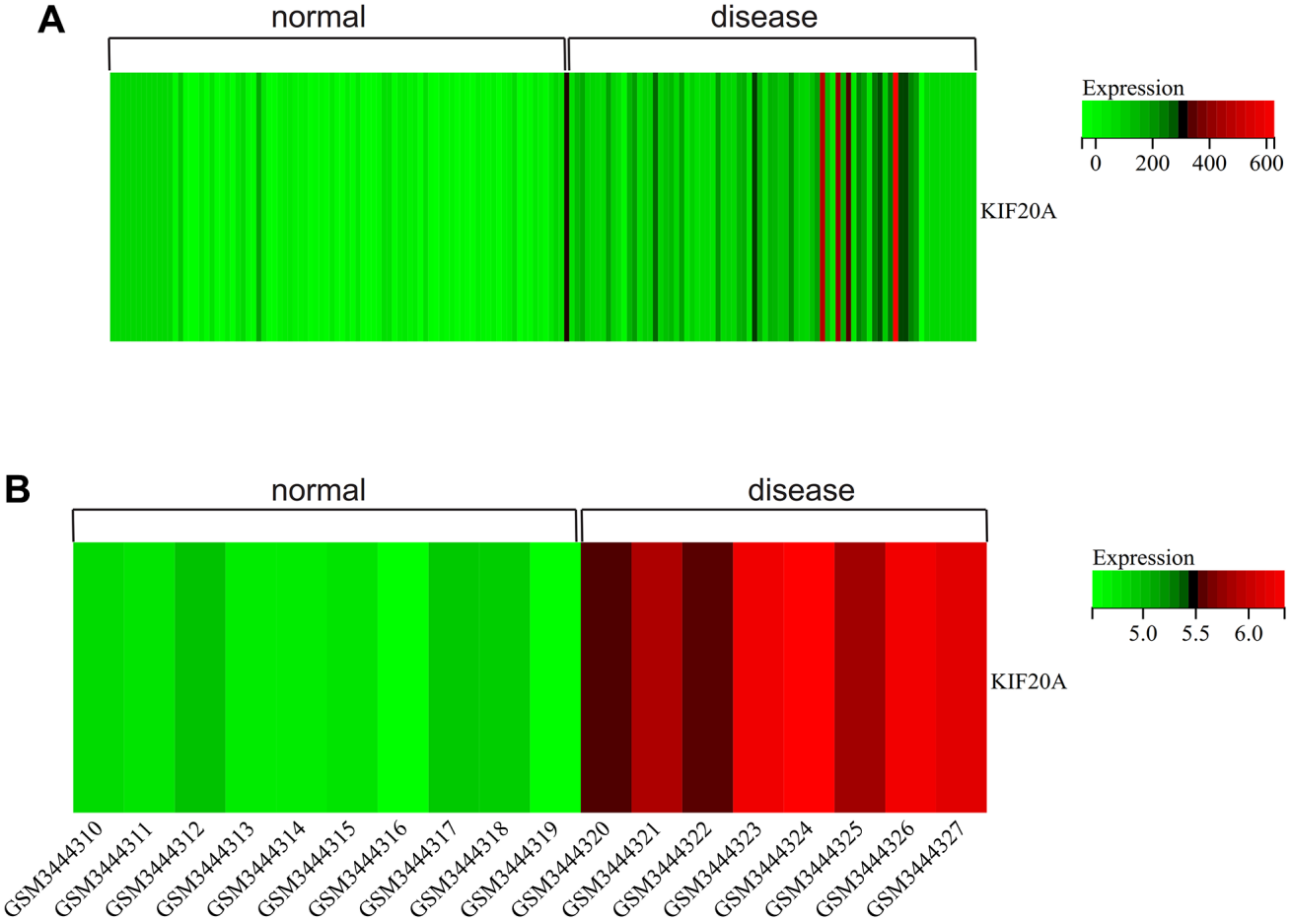
**Heatmap.** (**A**) GSEGSE14762, GSE53757. (**B**) GSE121711.

We visualized the gene expression heatmap for core genes in the samples, with Figure 11A showing the merged matrix results of GSE14762 and GSE53757. The results showed that KIF20A was highly expressed in RCC (Figure 11A). The core gene heatmap in GSE121711 showed that KIF20A was highly expressed in BC tissue (Figure 11B). The KIF20A gene was highly expressed in the RCC and BC samples, while it was expressed at low levels in normal tissue samples, indicating its potential regulatory role in these cancers.

### Immune infiltration analysis

**Figure 12:**
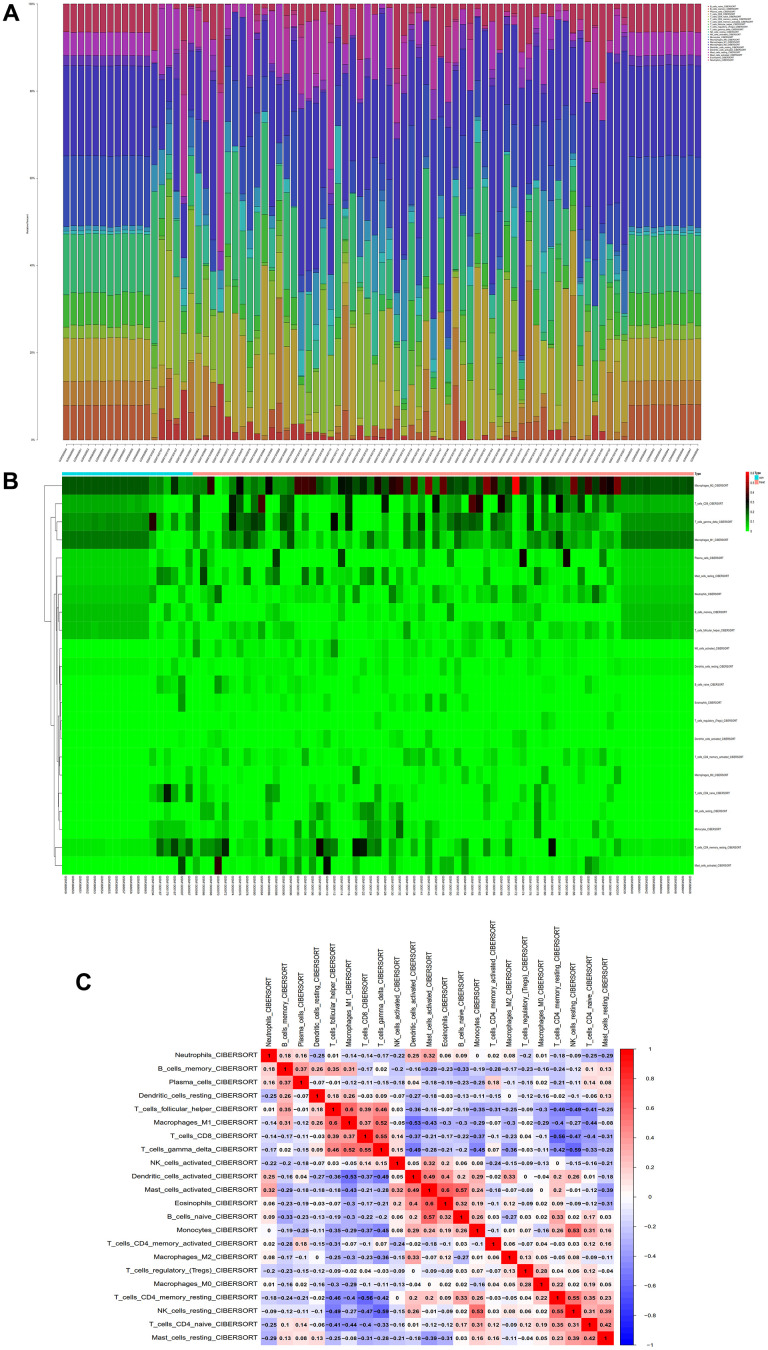
**Immunoinfiltration analysis.** (**A**) Whole-gene expression matrix results of proportions of immune cells. (**B**) Immune cell expression calorigram. (**C**) Plot of coexpression patterns between immune cell components.

We conducted immune infiltration analysis of the batch-corrected merged matrix of GSE14762 and GSE53757 using the CIBERSORT package and obtained the proportion of immune cells in the whole gene expression matrix at 95% confidence (Figure 12A), as well as the heatmap of immune cell expression from the dataset (Figure 12B). We also performed coexpression correlation analysis on the infiltrating immune cells and obtained a coexpression pattern map between immune cell components (Figure 12C).

### Role of KIF20A in the pRB Ser 780/CyclinA signaling pathway

**Figure 13:**
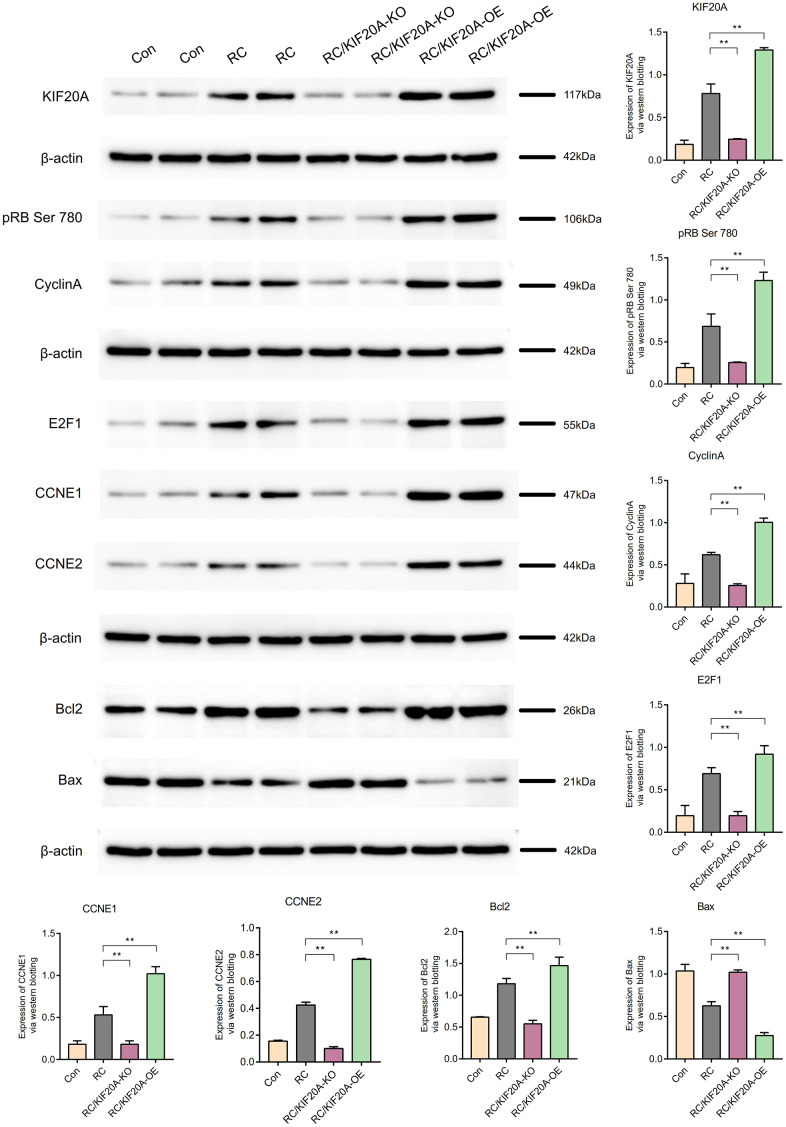
KIF20A might inhibit apoptosis in RCs via the pRB Ser 780/CyclinA signaling pathway.

KIF20A expression in the RC group was upregulated, as shown by western blotting. The core proteins (including pRB Ser 780, CyclinA, E2F1, CCNE1, and CCNE2) in the pRB Ser 780/CyclinA signaling pathway were also upregulated in the RC group. Bcl2 expression was high in the RC group, and Bax expression in the RC group was downregulated, which showed that apoptosis was inhibited in the RC group. When KIF20A was knocked down, the core proteins (including pRB Ser 780, CyclinA, E2F1, CCNE1, and CCNE2) in the pRB Ser 780/CyclinA signaling pathway were also downregulated in the RC/KIF20A-KO group, and apoptosis was enhanced. However, when KIF20A was overexpressed in the RC tissues, the core proteins (including pRB Ser 780, CyclinA, E2F1, CCNE1, and CCNE2) in the pRB Ser 780/CyclinA signaling pathway were also upregulated in the RC/KIF20A-OE group, and apoptosis was inhibited. The results showed that KIF20A might inhibit apoptosis in RC via the pRB Ser 780/CyclinA signaling pathway (Figure 13).

## DISCUSSION

Muscle-invasive bladder cancer is more aggressive and can spread to other parts that affect the urinary system [15, 16]. Kidney cancer, on the other hand, occurs when cells in the kidney grow uncontrollably. Both bladder cancer and kidney cancer can be harmful, especially if not detected and treated early [17]. If the cancer is allowed to grow and spread, it can lead to more severe symptoms and can even be life-threatening. Studying bladder and kidney cancers is very important. This research revealed that KIF20A was highly expressed in bladder and kidney cancers, and the higher the expression of KIF20A was, the worse the prognosis was [18–20].

KIF20A (Kinesin Family 20A) is a protein that plays a role in cell division and is involved in the formation of the mitotic spindle during cell division. Several studies have investigated the relationship between KIF20A and bladder cancer [21, 22]. Research suggests that KIF20A is overexpressed in bladder cancer cells, meaning that there is an abnormally high amount of the protein in these cells [23, 24]. Overexpression of KIF20A has been associated with increased tumor growth, invasion, and metastasis in bladder cancer [25]. Another study found that KIF20A was involved in the resistance of bladder cancer cells to chemotherapy drugs. The study showed that inhibiting KIF20A expression enhanced sensitivity to chemotherapy [26]. Overall, these studies suggest that KIF20A might affect the progression of bladder cancer [27].

KIF20A may be involved in the progression of various types of cancer, including renal cancer [28]. Renal cancer is usually diagnosed in its early stages, but it can be challenging to treat if it has spread to other parts of the body [29]. Studies have shown that KIF20A is overexpressed in renal cancer cells compared to normal kidney cells. Overexpression of KIF20A has been associated with aggressive behavior and poor prognosis in renal cancer patients. In addition, inhibiting KIF20A expression has been shown to reduce renal cancer cell proliferation and to induce cell death, suggesting that KIF20A may be a potential therapeutic target for renal cancer. Overall, the relationship between KIF20A and renal cancer suggests that KIF20A may play a critical role in development, and targeting KIF20A may be a potential strategy for the treatment of renal cancer [30].

KIF20A is overexpressed in many types of cancer cells, including breast, lung, liver, pancreatic, ovarian, and colorectal cancers, as well as bladder and renal cancers, as we discussed earlier [31]. Overexpression of KIF20A has been associated with aggressive behavior and poor prognosis in cancer patients, and inhibiting KIF20A expression has been shown to reduce cancer cell proliferation and induce cell death *in vitro* and in animal models [32]. Moreover, studies have shown that KIF20A interacts with other proteins involved in cell cycle regulation and cancer development, such as Aurora-A and PLK1, suggesting that KIF20A may play a crucial role in the regulation of cell division and cancer progression. Overall, the relationship between KIF20A and cancer suggests that KIF20A may be a potential therapeutic target for cancer treatment [33]. However, further research is needed to fully understand the underlying mechanisms of KIF20A in cancer and to develop effective therapeutic strategies targeting this protein [34].

The limitations of this study are as follows: Other factors, such as age, sex, lifestyle, and genetic background, can also influence the development and progression of cancer, and it can be challenging to control for these factors in studies.

The future research prospects are as follows: In future studies, we should include more patients with bladder and kidney cancer in the study population and then use multiple experimental techniques to detect KIF20A expression in these patients. Artificial intelligence or machine learning algorithms have been used to establish associations between KIF20A expression and survival outcomes in patients with bladder and kidney cancers [35, 36].

## CONCLUSIONS

KIF20A is overexpressed in bladder cancer and renal cell carcinoma and may play a role in the development and progression of these cancers. KIF20A may be used as a molecular target for early diagnosis and precise treatment of bladder cancer and renal cell carcinoma and to provide a basis for the study of the mechanism of bladder cancer and renal carcinoma.
